# Anti-inflammatory Effects of Curcumin in Microglial Cells

**DOI:** 10.3389/fphar.2018.00386

**Published:** 2018-04-20

**Authors:** Yangyang Yu, Qian Shen, Yihong Lai, Sun Y. Park, Xingmei Ou, Dongxu Lin, Meiling Jin, Weizhen Zhang

**Affiliations:** ^1^Shenzhen University Health Science Center, Shenzhen, China; ^2^Bio-IT Fusion Technology Research Institute, Pusan National University, Busan, South Korea

**Keywords:** curcumin, neuroinflammation, TLR2, HO-1, microglial cells

## Abstract

Lipoteichoic acid (LTA) induces neuroinflammatory molecules, contributing to the pathogenesis of neurodegenerative diseases. Therefore, suppression of neuroinflammatory molecules could be developed as a therapeutic method. Although previous data supports an immune-modulating effect of curcumin, the underlying signaling pathways are largely unidentified. Here, we investigated curcumin’s anti-neuroinflammatory properties in LTA-stimulated BV-2 microglial cells. Inflammatory cytokine tumor necrosis factor-α [TNF-α, prostaglandin E2 (PGE2), and Nitric Oxide (NO] secretion in LTA-induced microglial cells were inhibited by curcumin. Curcumin also inhibited LTA-induced inducible NO synthases (iNOS) and cyclooxygenase-2 (COX-2) expression. Subsequently, our mechanistic studies revealed that curcumin inhibited LTA-induced phosphorylation of mitogen-activated protein kinase (MAPK) including ERK, p38, Akt and translocation of NF-κB. Furthermore, curcumin induced hemeoxygenase (HO)-1HO-1 and nuclear factor erythroid 2-related factor 2 (Nrf-2) expression in microglial cells. Inhibition of HO-1 reversed the inhibition effect of HO-1 on inflammatory mediators release in LTA-stimulated microglial cells. Taken together, our results suggest that curcumin could be a potential therapeutic agent for the treatment of neurodegenerative disorders via suppressing neuroinflammatory responses.

## Introduction

Chronic neuroinflammation plays an important role in various neurodegenerative diseases, including AD, Parkinson’s disease (PD), Huntington’s disease (HD), stroke, amyotrophic lateral sclerosis (ALS), and multiple sclerosis (MS) (Spangenberg and Green, 2017). Neuroinflammation is interceded by the activation of microglia, the prime effector cells and resident immune cells of the CNS (Nakagawa and Chiba, 2015). Microglial cells can be activated in response to neuronal death or neuronal damage induced by neuroinflammatory responses or by extracellular toxins, such as bacteria and pathogens (Larochelle et al., 2015). In neuroinflammation, activated microglia releases various kinds of cytokines, chemokines, reactive oxygen species, and reactive nitrogen species for the development and maintenance of inflammatory responses (Moss and Bates, 2001). Excessive production of these inflammatory mediators could cause neuronal damage and death. Accumulated evidence suggests that control of microglial activation could attenuate the severity of neurodegenerative disease (Perry et al., 2010). Therefore, the development of anti-neuro-inflammatory agents for the inhibition of microglial activation could be beneficial for the treatment of neurodegenerative diseases.

Microglia express pattern recognition receptors (PRR) that can bind to pattern-associated molecular patterns (PAMPs) and damage-associated molecular patterns (DAMPs) such as lipopolysaccharide (LPS) and lipoteichoic acid (LTA), respectively (Jack et al., 2005). TLRs, a major class of PRRs, play a crucial role in host defense by inducing innate immune responses. Increasingly, studies have indicated that TLR2 agonist LTA is involved in the pathogenesis of CNS infectious diseases and can induce neuronal damage (Neher et al., 2011). Inhibition of TLR2 activation attenuates microglial cell activation and amyloid β accumulation in the brain (McDonald et al., 2016; Hossain et al., 2017). Signal transduction via TLR2 is mediated by different adaptor proteins, including MyD88, which promotes downstream signaling via MAPK and NF-κB activation leading to the expression of inflammatory mediators (Larochelle et al., 2015).

Inflammatory and oxidative molecules are very potent activators of Keap-Nrf2 (NF-E2-related factor 2), which induces the expression of Phase II detoxification enzymes to adapt to the oxidative stress condition (Rojo et al., 2010). Usually, Nrf2 acts in an inactive form. Upon stimulation, Nrf2 separates from Keap1 and translocates into the nucleus, where it binds to the antioxidant response element (ARE) to activate the transcription of antioxidant genes for cytoprotection (Ma, 2013; Cho et al., 2015). One of the Nrf2-regulated genes is heme oxygenase-1 (HO-1), which has an ARE sequence in its promoter region. Recently, HO-1 has been reported to be a predominant factor in controlling oxidative stress and inflammatory responses in neurodegenerative diseases (Schipper et al., 2009). HO-1 is the first inducible rate-limiting enzyme in the degradation of heme into by-products. HO-1 may provide neuroprotection or neurotoxic effect because of the balance between the beneficial and toxic effects of heme and heme products (Mancuso et al., 2010). One by-product of HO-1, Bilirubin, has been demonstrated to protect neurons from oxidative stress *in vivo* and *in vitro*. Bilirubin can be oxidized to biliverdin by scavenging peroxyl radicals (Chen, 2014). It has been suggested that HO-1, biliverdin, and CO have anti-inflammatory properties (Jazwa and Cuadrado, 2010). Another study has suggested that mice lacking HO-1 were vulnerable to pro-inflammatory stimuli and developed chronic inflammation due to reduced iron levels (Chora et al., 2007). Furthermore, a recent study suggested that up-regulation of the Nrf2 and HO-1 pathways significantly inhibited the inflammatory reaction in activated microglia (Kim et al., 2016). Nrf2 inhibited microglial hyperactivation by suppressing p38 MAPK and the NF-κB signaling pathway (Kim B.W. et al., 2013). Knockdown of Nrf2 in mice was shown to be hypersensitive to neuroinflammation, as indicated by an increase in the inflammatory markers iNOS, IL-6, and TNF-α (Rojo et al., 2010). Consequently, Nrf2 and HO-1 have been considered as important therapeutic targets for neurodegenerative diseases (Koh et al., 2011; Zhang et al., 2014).

Curcumin, the main curcuminoid isolated from *Curcuma longa* L. (turmeric) has been used for centuries in Southeast Asia both as a medicinal remedy and as food (Kunnumakkara et al., 2017). Curcumin, demethoxycurcumin, bisdemethoxycurcumin, ar-turmerone, α-turmerone, and β-turmerone are the major bioactive compounds found in *C. longa.* In modern pharmacological studies, *C. longa* constituents, particularly curcumin, has shown promising pharmacological activities due to its anti-neuroinflammatory, neuroprotective, chemopreventive, immunomodulatory, and potentially chemotherapeutic effects (Garcia-Alloza et al., 2007; Zhou et al., 2017). A previous study showed that curcumin inhibited LPS-induced inflammatory responses in RAW264.7 macrophages, suggesting a potential role of curcumin in anti-Gram-negative bacterial infection (Zhou et al., 2017) and both *in vivo* and *in vitro* research have shown that curcumin exhibits anti-inflammatory effects (Garcia-Alloza et al., 2007; Prakobwong et al., 2011; Parada et al., 2015; Li et al., 2016). Furthermore, curcumin has also been reported to promote the development of the M2 microglial phenotype in an HO-1-dependent manner and reduce iNOS induction, protecting microglial cells against oxidative stress (Parada et al., 2015). In the present study, we investigated whether curcumin could affect LTA-induced microglial activation. The TLR2 ligand LTA is a major constituent of the cell wall of Gram-positive bacteria. We show that curcumin exhibits anti-inflammatory and antioxidant effects in LTA-stimulated BV2 microglia through activation of HO-1/Nrf2/ARE cytoprotective mechanisms.

## Materials and Methods

### Materials

Curcumin and other reagents were purchased from Sigma (C7727, >80%, St. Louis, MO, United States). Protoporphyrin IX (SnPP) and antibodies directed against HO-1 (sc-390991) - Nrf2 (sc-722), TATA-binding protein (TBP; sc-74595), α-tubulin (sc-134237), and β-actin (sc-130065) - were purchased from Santa Cruz Biotechnology, Inc., (Dallas, TX, United States). Antibodies directed against iNOS (13120) - phosphorylated (p)-MAPK (9910s), MAPK (9926), protein kinase B (Akt; 4685), p-Akt (13038), and an NF-κB pathway kit (9936) - were purchased from Cell Signaling Technology, Inc., (Danvers, MA, United States). LTA was obtained from InvivoGen (tlrl-pslta,Toulouse, France). Additionally, JNK inhibitor (JNK inhibitor II; 420119), Akt inhibitor (wortmannin; 12-338), ERK inhibitor (PD98059, 513000), and p38 inhibitor (SB230580, 559395) were purchased from EMD Millipore (Billerica, MA, United States). The cell culture medium, DMEM, and fetal bovine serum (FBS) were purchased from Gibco BRL (now Invitrogen Corporation, Carlsbad, CA, United States).

### Cell Culture

Mouse BV-2 microglial cells were purchased from ATCC. Cells were cultured in DMEM supplemented with 10% heat-inactivated FBS and 0.1% penicillin-streptomycin (BioSource International, Camarillo, CA, United States) at 37°C in a humidified atmosphere of 5% CO_2_ and 95% air.

### Cell Viability Assay

The cytotoxicity of curcumin was assessed using a microculture [3-(4,5-Dimethylthiazol-2-yl)-2,5-diphenyltetrazolium bromide] (MTT)-based colorimetric assay. Cells were incubated in 24-well plates at a density of 5 × 10^5^ cells per well. The MTT solution (5 ml of 5 mg/ml) was added to each well (final concentration 62.5 mg/ml). After incubation for 3 h at 37°C in 5% CO_2_, the supernatant was removed and the formazan crystals produced in viable cells were solubilized with 150 ml of dimethylsulfoxide (DMSO). The absorbance of each well was then read at 570 nm using a microplate reader (Wallac 1420; PerkinElmer, Inc., Boston, MA, United States).

### Measurement of Nitrite Concentration

NO synthesis in cell cultures was measured by Griess method with microplate. To measure nitrite, 100-μl aliquots were removed from conditioned medium and incubated with an equal volume of the Griess reagent [1% sulfanilamide/0.1%N-(1-naphthyl)-ethylenediaminedihydrochloride/2.5% H_3_PO_4_] at room temperature for 10 min. The nitrite concentration was determined by measuring the absorbance at 540 nm with a Vmax 96-well microplate spectrophotometer (Molecular Devices, Menlo Park, CA, United States). Sodium nitrite was used as a standard.

### Measurement of TNF-α and PGE_2_ Concentration

The cells were incubated first with various concentrations of curcumin for 1 h and then with LTA for 16 h. Following 24 h incubation, TNF-α and PGE_2_ levels were quantified in the culture media using an enzyme-linked immunosorbent assay (ELISA) kit (R&D Systems, Minneapolis, MN, United States) according to the manufacturer’s instructions.

### Preparation of Nuclear Extract

BV-2 microglial cells were washed three times with cold PBS and collected in 3000 μl PBS using centrifugation at 800 ×*g* for 5 min (4°C). The cell pellets were suspended in buffer A [10 mM HEPES-KOH (pH 7.9); 1.5 mM MgCl_2_; 10 mM KCl; 0.5 mM dithiothreitol (DTT); 0.2 mM protease inhibitor (PI)] and incubated for 5 min on ice. Buffer B [10 mM HEPES-KOH (pH 7.9); 1.5 mM MgCl_2_; 420 mM NaCl; 0.2 mM EDTA; glycerol 25% v/v; 0.1 mM DTT; 0.2 mM PI] was added to the cell extract and was incubated on ice for 5 min prior to centrifugation at 11,000 ×*g* for 1 min at 4°C. Nuclear proteins were extracted with the addition of complete lysis buffer B [10 mM HEPES-KOH (pH 7.9); 1.5 mM MgCl_2_; 10 mM KCl; 0.5 mM DTT; 0.2 mM PI; 25% (w/v) glycerin; 420 mM NaCl; 0.2 mM EDTA] for 30 min at 4°C with occasional vortexing. Following centrifugation at 11,000 ×*g* for 5 min at 4°C, the supernatants were collected and stored at -70°C.

### Western Blot Analysis

BV-2 cells were harvested in an ice-cold lysis buffer (1% Triton X-100; 1% deoxycholate; 0.1% sodium dodecyl sulfate). The protein content of the cell lysates was subsequently determined using Bradford reagent (Bio-Rad Protein Assay Kit I5000001; Bio-Rad Laboratories, Inc., Hercules, CA, United States). Total proteins in each sample (50 μg) were separated by 7.5% SDS-PAGE and transferred to polyvinylidene difluoride membranes. Following blocking of the non-specific binding sites with 5% non-fat milk at room temperature for 30 min, the membranes were incubated with primary antibodies directed against iNOS (1:500), p-Akt (1:1,000), p-MAPK (1:1,000), MAPK (1:1,000), p-p65, p65 (1:500), p-IκBα, IκBα (1:1,000), HO-1 (1:1,000), Nrf2 (1:1,000), TBP (1:3,000), α (1:1,000), HO-1 (1:1.0), and actin (1:3,000) for 16 h at 4°C. This was followed by incubation with horseradish peroxidase-conjugated anti-rabbit (sc-2768; 1:5,000) or anti-mouse (sc-2371; 1:5,000) secondary antibodies (Santa Cruz Biotechnology, Inc.) at room temperature for 1 h. Tubulin was used as the loading control for each lane. The proteins were visualized using an enhanced chemiluminescence detection kit (GE Healthcare, Chicago, IL, United States). Following washing with PBS with Tween-20, the protein bands were visualized using the Gel Docsed as the loading control for each lane. The proteins were visualized using Quant 350 analyzer (GE Healthcare).

### Real-Time RT-PCR

Total RNA was isolated from cells using an RNA spin miniRNA isolation kit (GE Healthcare, Uppsala, Sweden) according to the manufacturer’s instructions. cDNA was synthesized from 1 μg of total RNA using Maxime RT PreMix (Takara, Gyeonggi-do, Japan) and anchored oligo-dT15-primers. Real-time PCR was performed using a Chromo4^TM^ instrument (Bio-Rad) and SYBR Green Master Mix (Applied Biosystems, Foster City, CA, United States). Relative amounts of target mRNA were determined using the comparative threshold (Ct) method by normalizing target mRNA Ct values to those for β-actin (Ct). Prime sequences used in the study were showed in **Table 1**.

**Table 1:** Name and sequence of primers used for reverse transcription-quantitative polymerase chain reaction.

Gene name	Primer sequence (5′–3′)	Gene ID	Product size, bp
Inducible nitric oxide synthase	F: GGCACCGAGATTGGAGTTC	NM001313921	174
	R: GGTCACATTCTGCTTCT		
Cyclooxygenase-2	F: TCAGGTCATTGGTGGAGAGG	NM011198.4	150
	R: ATGGTGGCATACATCATCAGAC		
Heme oxygenase-1	F: AGGTCCTGAAGAAGATTGC	NM010442.2	175
	R: TCTCCAGAGTGTTCATTCG		
β-actin	F: GCACCACACCTTCTACAA	NM007393.5	156
	R: TACGACCAGAGGCATACA		


*F, forward; R, reverse*.

### Statistical Analysis

Data are expressed as the mean (standard deviation, SD). Each experiment was repeated at least three times. Statistical analysis was performed using the Statistical Package for GraphPad Prism software (version 16.0) to determine significant differences. We used either Student’s *t*-test or one-way analysis of variance (ANOVA) followed by Dunn’s *post hoc* tests for analyses. *P*-values < 0.05 were considered statistically significant.

## Results

### Curcumin Did Not Affect Cell Viability

**FIGURE 1:**
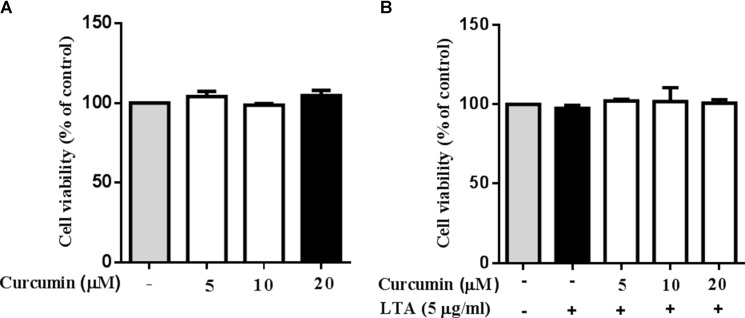
Effect of curcumin on BV-2 microglial cell viability. **(A)** BV2 cells were treated with various concentrations of curcumin (5, 10, and 20 μM) for 24 h. **(B)** BV2 cells were preincubated with curcumin (5, 10, and 20 μM) for 1 h, and then exposed to LTA (5 μg/ml) for 24 h. Cell viability was measured by MTT assay. Statistical significance was determined by one-way ANOVA. All data were mean ±*SD* of three experiments.

Cell viability experiments were carried out to determine whether concentrations of curcumin used in this study affected the viability of BV2 microglia. **Figure 1** shows that curcumin at the concentration range of 5–20 μM, together with or without 5 μg/ml LTA, did not produce cytotoxicity in BV2 microglia. Therefore, we used these concentrations of curcumin for further study.

### Curcumin Prevented the Production of Neuroinflammatory Molecules in LTA-Activated BV2 Microglia

**FIGURE 2:**
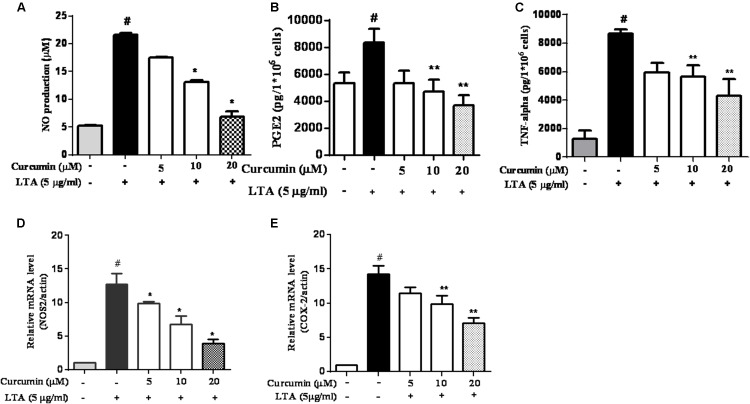
Curcumin inhibited neuroinflammatory mediators release from LTA-stimulated BV-2 microglial cells. Cells were treated with different concentrations of curcumin (5, 10, and 20 μM) for 1 h, then incubated with LTA (5 mg/ml) under serum-free conditions. **(A)** After 16 h of stimulation, nitrite content was measured using the Griess reaction. **(B,C)** The concentration of PGE2 and TNF-α, in the culture media was measured using a commercial enzyme-linked immunosorbent assay (ELISA) kit. **(D,E)** Cells were treated with different concentrations of curcumin (5, 10, and 20 μM) for 1 h then incubated with LTA (5 μg/ml) under serum-free conditions. After 4 h of stimulation, the mRNA expression levels of iNOS and COX-2 were determined by qRT-PCR. Statistical significance was determined by one-way ANOVA. Each bar represented the mean (SD) from three independent experiments per group. ^#^*P* < 0.01 vs. negative control, ^∗^*P* < 0.05, ^∗∗^*P* < 0.01 vs. the LTA-treated control.

To investigate the effects of curcumin on the secretion of inflammatory cytokines, BV2 cells were treated with LTA in the presence and absence of curcumin for 24 h. Curcumin was not removed before LTA addition. Release of NO, PGE2, and TNF-α were significantly and dose-dependently reduced by curcumin (**Figures 2A–C**). Furthermore, LTA increased the mRNA expression of iNOS and COX-2. Incubation with curcumin suppressed the mRNA expression of COX-2 and iNOS in BV2 microglial cells stimulated by LTA in a concentration-dependent manner (**Figures 2D,E**).

### Curcumin Suppressed LTA-Induced Activation of NF-κB in BV-2 Microglial Cells

**FIGURE 3:**
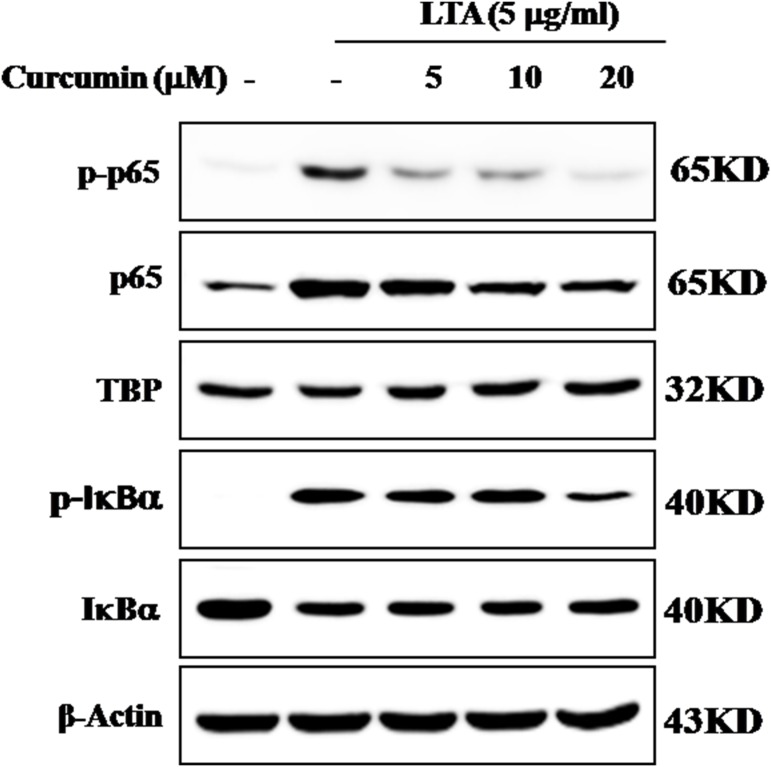
Inhibitory effects of curcumin on LTA-induced activation of NF-κB in BV2 cells. BV-2 microglial cells were treated with curcumin followed by LTA (5 μg/ml) treatment for 0.5 h. Nuclear translocation of (NF-κB) p65 was confirmed by western blotting. The cytosolic extracts were analyzed by western blotting with anti-IκB-α and anti-p-IκB-α antibodies. For western blot detection of TBP, α-tubulin was used as a protein-loading control for each lane.

The genes encoding inflammatory protein expression in response to microglial activation were under the transcription control of NF-κB. Therefore, we examined the effect of curcumin on the activation of NF-κB in LTA-stimulated microglial cells. The results showed that LTA induced a characteristic increase in the phosphorylation of IκBα. Following pre-treatment with curcumin, levels of p-IκBα were significantly reduced in a concentration-dependent manner (**Figure 3** and Supplementary Figure S1). Consistently, the nuclear translocation of the NF-κB p65 subunit induced by LTA was also attenuated by pre-treatment with curcumin. Taken together, curcumin likely attenuates the expression of neuroinflammatory molecules by suppressing the nuclear translocation and activation of NF-κB. Quantification with statistical analysis was provided as supporting data.

### Curcumin Inhibited LTA-Induced Activation of p38, and ERK MAPK in BV-2 Microglial Cells

**FIGURE 4:**
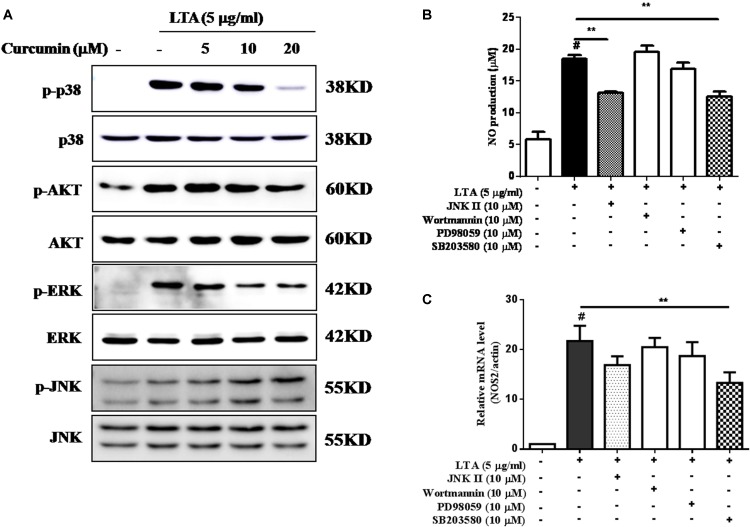
Curcumin inhibited LTA-induced phosphorylation of p38, ERK, and Akt in BV-2 microglial cells. **(A)** BV-2 microglial cells were treated with the indicated concentrations of curcumin for 1 h and then stimulated with LTA (5 μg/ml) for 1 h. An equal amount of cell extract was analyzed by western blotting with anti-p-ERK1/2, anti-p-c-Jun N-terminal kinase (JNK), anti-p-p38, and anti-p-Akt antibodies. ERK1/2, JNK, p38 and Akt bands indicated that the induction of total ERK1/2, JNK, p38, and Akt protein was not changed. BV-2 cells were treated with JNK inhibitor (JNK II, 10 mM), Akt inhibitor (Wor, 5 mM), ERK inhibitor (PD98059, 10 μM), or p38 inhibitor (SB230580, 10 μM) for 1 h, following treatment with LTA for 16 h. **(B)** Subsequently, the levels of NO production were determined. **(C)** The expression level of iNOS was also determined by qRT-PCR. Statistical significance was determined by Student’s *t*-test. Each bar represents the mean (SD) from three independent experiments per group. ^#^*P* < 0.01 vs. negative control, ^∗^*P* < 0.05, and ^∗∗^*P* < 0.01 vs. the LTA-treated group.

Apart from NF-κB, MAPKs are also upstream modulators of neuroinflammatory molecules in microglial cells. Previous studies showed that curcumin antagonized LPS-induced MAPKs phosphorylation in microphage (Yang et al., 2008; Kunnumakkara et al., 2017). To investigate whether curcumin inhibits neuroinflammation through regulating MAPKs, we examined its effects on LTA-induced MAPK phosphorylation. BV-2 microglial cells were pre-treated with different concentrations of curcumin for 3 h and were then stimulated with LTA for 1 h. As shown in **Figure 4A** and Supplementary Figure S2, curcumin inhibited LTA-induced ERK, p38, and Akt phosphorylation. However, up to 20 μM curcumin did not affect LTA-induced JNK phosphorylation. MAPKs pathway have been reported to mediate the production of cytokines, chemokine, and other neuroinflammatory molecules. Therefore, we next investigated the role of ERK, p38, JNK, and Akt in BV2 cells’ neuroinflammatory molecule production using the ERK, p38, JNK, and Akt inhibitors. However, only the p38 inhibitor SB203580 significantly decreased LTA-induced release of NO and mRNA expression levels of iNOS (**Figures 4B,C**). Although phosphorylation of JNK was not inhibited by curcumin, the JNK inhibitor II significantly inhibited LTA-induced NO release (**Figure 4B**). The results suggest that MAPKs’ signaling pathways are involved in curcumin’s anti-neuroinflammatory effects in LTA-stimulated microglial. Quantification with statistical analysis is provided as supporting data.

### Inhibition of HO-1 Signaling Abolished Curcumin’s Inhibitory Effect on Neuroinflammatory Responses

**FIGURE 5:**
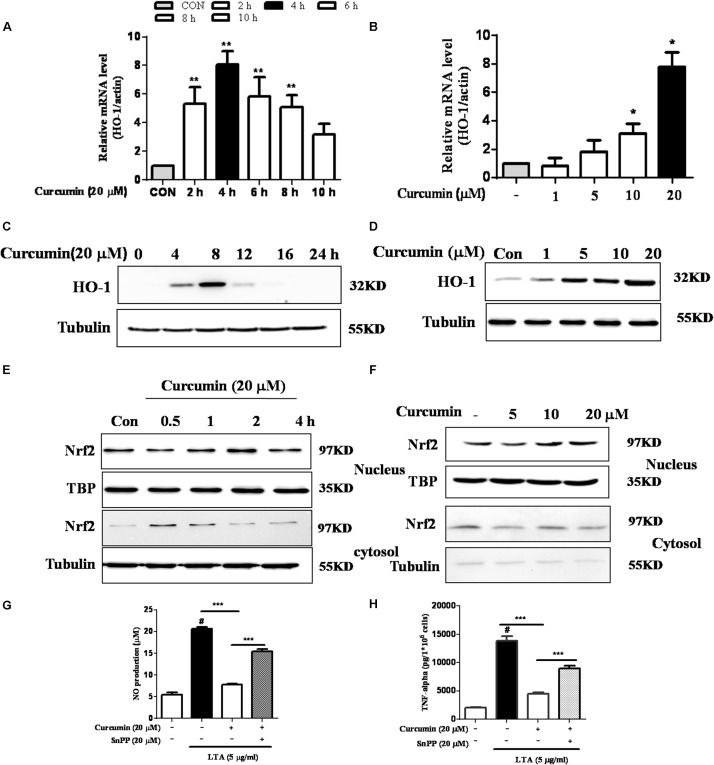
Effects of HO-1 on curcumin-mediated anti-neuroinflammatory effects in LTA-stimulated microglial cells. **(A,B)** Cells were cultured with increasing concentrations of curcumin for 4 h or 20 μM of curcumin for the indicated times. mRNA expression level of HO-1 was determined by qRT-PCR. **(C,D)** Cells were cultured with increasing concentrations of curcumin for 8 h or 20 μM of curcumin for the indicated times. HO-1 protein expression was determined by western blot. **(E,F)** Cells were incubated with 20 μM curcumin for the indicated time or were incubated with the indicated concentration of curcumin for 1 h. Nuclear localization of Nrf2 was determined by western blot. TBP was used as a protein loading control for each lane. **(G,H)** The cells were incubated with curcumin for 1 h and then exposed to LTA with or without the HO-1 inhibitor SnPP (20 μM, HO-1 inhibitor) for 16 h. The secretion of NO and TNF-α were determined. Statistical significance was determined by Student’s *t*-test. Each bar represents the mean (SD) from three independent experiments per group. ^#^*P* < 0.01 vs. negative control, ^∗∗^*P* < 0.01, ^∗∗∗^*P* < 0.001 vs. the LTA-treated group.

HO-1 acts as an anti-inflammatory and antioxidant modulator in microglia (Schipper et al., 2009). Western blot and RT-PCR analyses showed that curcumin upregulated HO-1 expression at the protein and mRNA levels, as shown in **Figures 5A–D** and Supplementary Figure S3. The expression of HO-1 mRNA and protein were maximally increased in BV-2 microglia cells treated with 20μM curcumin for 4 h and 8 h respectively. Furthermore, curcumin increased Nrf2 nuclear translocation within 1 h and prolonged its nuclear translocation state to 2 h (**Figures 5E,F** and Supplementary Figure S3). Next, we investigated whether curcumin-induced HO-1 mediated an anti-neuroinflammatory response in LTA-stimulated BV-2 microglial cells. We treated cells with the HO-1 inhibitor SnPP. We then evaluated curcumin’s effect on LTA-induced NO and TNF-α release. Treatment with SnPP significantly suppressed curcumin-mediated inhibition of NO and TNF-a release (**Figures 5G,H**). Taken together, these results reveal that curcumin-dependent HO-1 and Nrf-2 signal activation plays a crucial role in downregulating neuroinflammatory responses. Quantification with statistical analysis is provided as supporting data.

## Discussion

Microglia, the major resident macrophages of the CNS, has been reported to be the main effector cells in mediating neuroinflammation and selective neuronal death (Perry et al., 2010). Microglial cells increase the production of neuroinflammatory molecules after exposure to activators such as LPS and LTA via their surface receptors, TLR4 and TLR2, respectively (Perry and Holmes, 2014; Hossain et al., 2017). Increased expression and activation of TLR2 is associated with the progression of neurodegenerative diseases, such as PD and dementia (Dzamko et al., 2017). For example, activation of TLR2 could upregulate α-synuclein in PD brains and play important roles in the pathogenesis of PD brains (Roodveldt et al., 2013). In addition, Kim C. et al. (2013) also showed that neurodegeneration was attenuated by either knockout or knockdown of TLR2 in rodent PD models. Thus, controlling TLR2-mediated microglia activation and neurotoxicity has been suggested as an important therapeutic approach to treating neurodegenerative diseases. A potential agent in this process could be curcumin, which has been shown to exert neuro-protective and anti-inflammatory effects in various experiment models (Parada et al., 2015; Li et al., 2016). Curcumin is a highly lipophilic natural compound. A previous study has well demonstrated that curcumin is able to cross the blood–brain barrier and that it is mainly concentrated in the hippocampus in the brain (Tsai et al., 2011). Some studies reported that curcumin inhibited HIV-1 gp120-induced neuronal damage and provided anti-neuroinflammtory effects in LPS-induced microglia (Gong et al., 2012). This protective effect of curcumin seems to be dependent on its anti-inflammatory actions. Curcumin could protect neurons against microglia-mediated neurotoxicity, while becoming inefficient under microglia-depleted conditions (Park et al., 2001; Yang et al., 2008; Parada et al., 2015). Similar studies in peripheral cells also showed the anti-inflammatory effects of curcumin. Using RAW 264.7 murine macrophages, studies have shown that curcumin inhibited PGE_2_, NO, and TNF-α release following LPS stimulation (Pae et al., 2008). However, the effects of curcumin on TLR2-induced neuroinflammation in microglial cells are not fully understood.

Regulation of the signaling pathways in activated microglia is important in maintaining CNS homeostasis, because deregulated neuroinflammatory responses can result in the death of adjacent neurons through the release of inflammatory molecules, such as cytokines, chemokines, NO, and ROS (Perry and Holmes, 2014; Spangenberg and Green, 2017). For example, excessive NO synthesis under endotoxins results in the formation of reactive nitrogen species and neuronal cell death (Perry et al., 2010). PGE_2_ has also been shown to contribute to the neuronal death through activation of the MAPK/ERK pathway in microglia (Xia et al., 2015). In this present study, we showed that curcumin inhibited the secretion of inflammatory mediators TNF-α, NO, and PGE_2_, and expression of iNOS and COX-2 in BV2 microglia stimulated with LTA. We further showed that curcumin attenuated these effects of LTA without altering cell survival, suggesting that curcumin is safe and could be considered as a potential therapeutic agent in neuroinflammation.

NF-κB is a main transcription factor which plays critical roles in regulating redox homeostasis. NF-κB is considered the master regulator of microglial inflammatory responses to neuronal injury (Acharyya et al., 2007). Recent studies showed that NF-κB activation controlled the expression of inflammatory molecules, such as NO, PGE_2_, and TNF-α, and IL-1b production (Acharyya et al., 2007). Therefore, modulation of NF-κB activation is considered a critical way to control microglial activation. The activation of the NF-κB signaling pathway is mediated by the IκB protein. The phosphorylation of IκB results in NF-κB dissociation, which leads to the induction of inflammatory mediators. In this study, it was shown that curcumin produced dual inhibition of phosphorylation and degradation of IκBα, as well as nuclear translocation of p65, suggesting that this agent could stabilize NF-κB in the microglial cytoplasm following stimulation with LTA in BV-2 microglial cells.

In mammalian cells, MAPKs signaling pathways, including ERK, JNK, and p38, contribute to the production of a wide variety of neuroinflammatory mediators (Chantong et al., 2014). In this present study, pre-treatment with curcumin decreased the phosphorylation of p38 and ERK. Furthermore, the p38 inhibitor SB203580 significantly reduced the secretion of NO and the mRNA expression of the key pro-inflammatory gene, iNOS. These results suggested that curcumin initiated the anti-neuroinflammatory effects in LTA-stimulated BV-2 microglial cells, partially through inhibition of p38 MAPK activation. The PI3K/Akt-dependent signaling pathway promotes inflammatory responses in microglia. The involvement of the Akt pathway has been shown in the expression of inflammatory mediators in microglia through the activation of NF-κB in microglia (Lo et al., 2015). Curcumin suppressed the phosphorylated Akt, the downstream target of PI3K. However, the PI3K inhibitor wortmannin did not show any inhibitory effect on the secretion of NO or the mRNA expression of iNOS. Taken together, these data suggest that the anti-neuroinflammatory effect of curcumin occurs mainly through inhibiting the NF-κB and MAPKs signaling.

We also identified the intracellular pathway that negatively regulates the inflammatory-molecule expression in microglial cells. Nrf2 is a redox-sensitive transcription factor that regulates microglial inflammatory responses to brain infections. The effect of Nrf2 has been described in different *in vivo* models where knockdown of Nrf2 in mice enhanced vulnerability to asthma or emphysema (Ma, 2013). Moreover, the TLR2/TLR4 agonist promoted inflammatory responses in Nrf2 KO mice compared to WT mice (Kong et al., 2011). In the current study, we showed that curcumin increased the expression of Nrf2 and its downstream protein HO-1. HO-1 is a key signaling molecule implicated in the regulation of inflammatory and oxidative responses. The HO-1 gene has an ARE sequence in its promoter region, which is a binding site for the transcription factor Nrf2. Several studies have proposed that NF-κB interrupts the Nrf-2-ARE signaling pathway, because many compounds that induced HO-1 and Nrf2 signaling incidentally repressed NF-κB activation (Li et al., 2016). HO-1 expression was essential for the microglial specific cytoprotective effect (Parada et al., 2015). Several studies have also shown an inverse correlation between HO-1 and inflammatory mediator secretion (Chora et al., 2007; Parada et al., 2015). In agreement, we observed that curcumin alone induced the expression of HO-1 in microglial cells. Furthermore, the HO-1 inhibitor abrogated curcumin anti-inflammatory effect in BV-2 microglial cells.

## Conclusion

**FIGURE 6:**
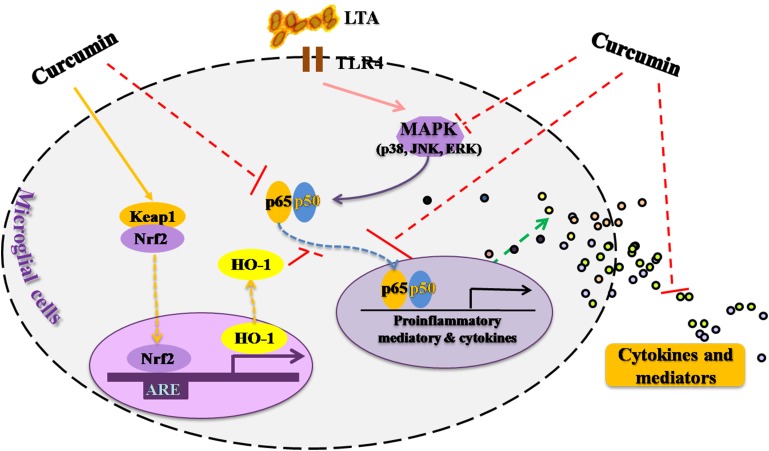
Anti-inflammatory mechanism of curcumin in LTA-stimulated microglial cells. Curcumin had anti-inflammatory activity in LTA-stimulated microglial cells through inhibiting NF-κB and p38 MAPK activation, and induced the expression of Nrf2 and HO-1.

This study demonstrated that curcumin had anti-inflammatory activity in LTA-stimulated microglial cells may through inhibiting NF-κB and p38 MAPK activation, and may induce the expression of Nrf2 and HO-1 (**Figure 6**). Furthermore, curcumin does not have cytotoxic effects in BV-2 microglial cells at its anti-inflammatory dose. Curcumin may have therapeutic potential for some neuroinflammation-associated disorders caused by Gram-positive bacteria.

## Author Contributions

YY led the experimental work. QS, YL, XO, DL, and MJ performed the experiments. MJ and SP analyzed the data. YY and MJ wrote the manuscript. YY and WZ obtained financial support, directed the study, and reviewed the manuscript.

## Conflict of Interest Statement

The authors declare that the research was conducted in the absence of any commercial or financial relationships that could be construed as a potential conflict of interest.

## Abbreviations

AD: Alzheimer’s disease
CNS: central nervous system
TNF-α: tumor necrosis factor-α
TLR: Toll-like receptor
MAPK: Mitogen-Activated Protein Kinase
JNK: c-Jun NH2-terminal protein kinase
ERK: extracellular signal-regulated kinase
NF-κB: nuclear factor-κB
PGE2: Prostaglandin E2
HO-1: hemeoxygenase-1
Nrf2: Nuclear factor erythroid 2-related factor 2
