# Supplementary material for: Anti-inflammatory Effects of Curcumin in Microglial Cells
Source: Front Pharmacol. 2018 Apr 20;9:386. doi: 10.3389/fphar.2018.00386 (PMC5922181; doi:10.3389/fphar.2018.00386)
Supplement: Supplementary file 1 [file Data_Sheet_1.docx]

**Supplementary Figures**

**S1. Inhibitory effects of curcumin on LTA-induced activation of NF-κB in BV2 cells.** BV-2 microglial cells were treated with curcumin followed by LTA (5 μg/ml) treatment for 0.5 h. Nuclear translocation of (NF-κB) p65 was confirmed by western blotting. The cytosolic extracts were analyzed by western blotting with anti-IκB-α and anti-p-IκB-α antibodies. For western blot detection of TBP and α-tubulin was used as a protein-loading control for each lane. Relative level of pp65, p65, p-iKBa and iKBa protein expression between curcumin untreated and treated cells were compared. Statistical significance was determined by t-test. ^##^P<0.01 vs. negative control, **P* < 0.05 , ** *P* < 0.01 and *** *P* < 0.001 vs. the LTA-treated but curcumin untreated group. All data were mean ±S.E.M of three experiments.

S2. Curcumin inhibited LTA-induced phosphorylation of p38, ERK, and Akt in BV-2 microglial cells. BV-2 microglial cells were treated with the indicated concentrations of curcumin for 1 h and then stimulated with LTA (5 μg/ml) for 1 h. An equal amount of cell extract was analyzed by western blotting with anti-p-extracellular signal-regulated kinase 1/2 (ERK1/2), anti-p-c-Jun N-terminal kinase (JNK), anti-p-p38 and anti-p-Akt antibodies. Relative level of phosphorylated protein expression between curcumin untreated and treated cells were compared. Statistical significance was determined by t-test. ^##^P<0.01 and ^###^P<0.001 vs. negative control, **P* < 0.05 , ** *P* < 0.01 and *** *P* < 0.001 vs. the LTA-treated but curcumin untreated group. All data were mean ±S.E.M of three experiments.

**S3. Effects of HO-1 on curcumin-mediated anti-neuroinflammatory effects in LTA-stimulated microglial cells.** Cells were cultured with increasing concentrations of curcumin for 8 h or 20 μM of curcumin for the indicated times. HO-1 protein expression was determined by western blot. Or cells were incubated with 20 μM curcumin for the indicated time or were incubated with the indicated concentration of curcumin for 1 h. Nuclear localization of Nrf2 was determined by western blot. TBP was used as a protein loading control for each lane. Relative level of HO-1 protein expression between untreated and treated cells were compared. Statistical significance was determined by t-test. **P* < 0.05 , ** *P* < 0.01 and *** *P* < 0.001 vs. control. All data were mean ±S.E.M of three experiments.
