# Supplementary figures and images for: Anti-inflammatory Effects of Curcumin in Microglial Cells
Source: Front Pharmacol. 2018 Apr 20;9:386. doi: 10.3389/fphar.2018.00386 (PMC5922181; doi:10.3389/fphar.2018.00386)

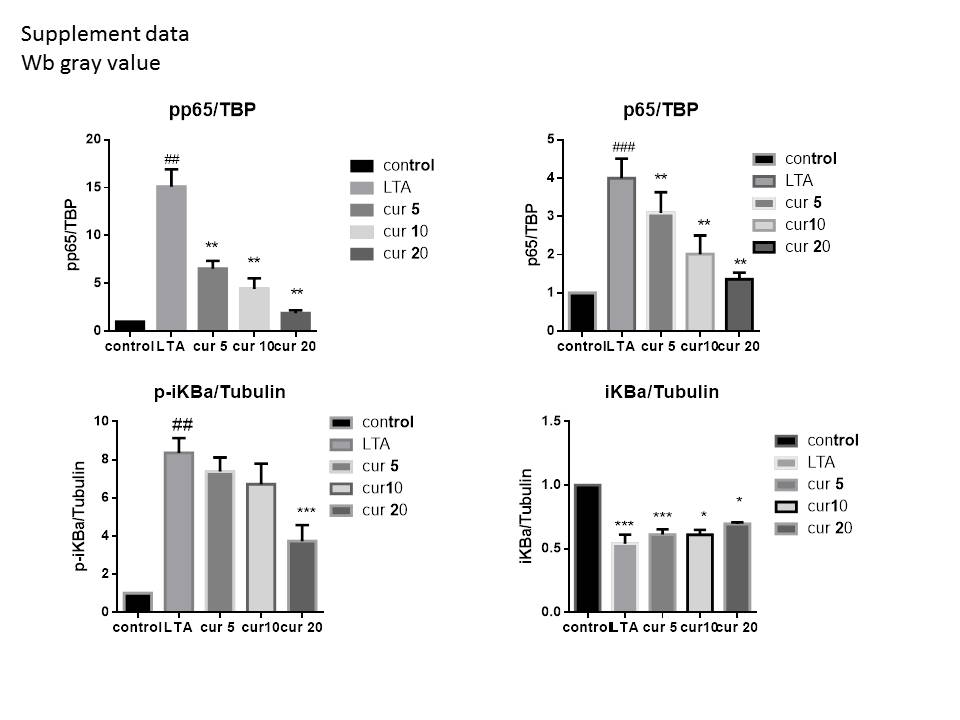

Supplement: Supplementary file 2 [file Image_1.JPEG]

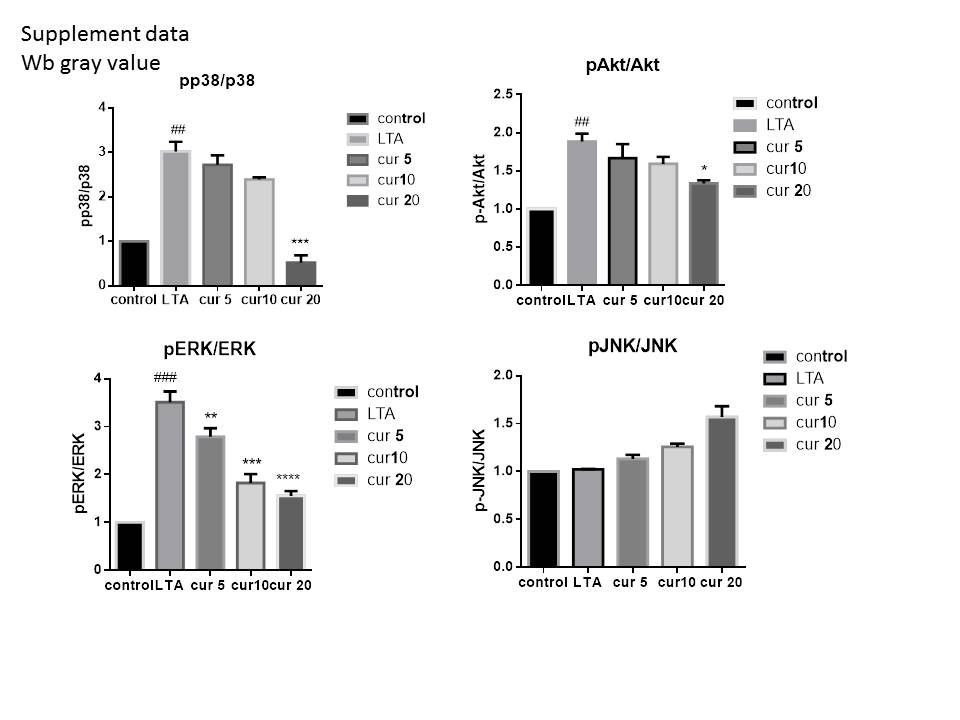

Supplement: Supplementary file 3 [file Image_2.JPEG]

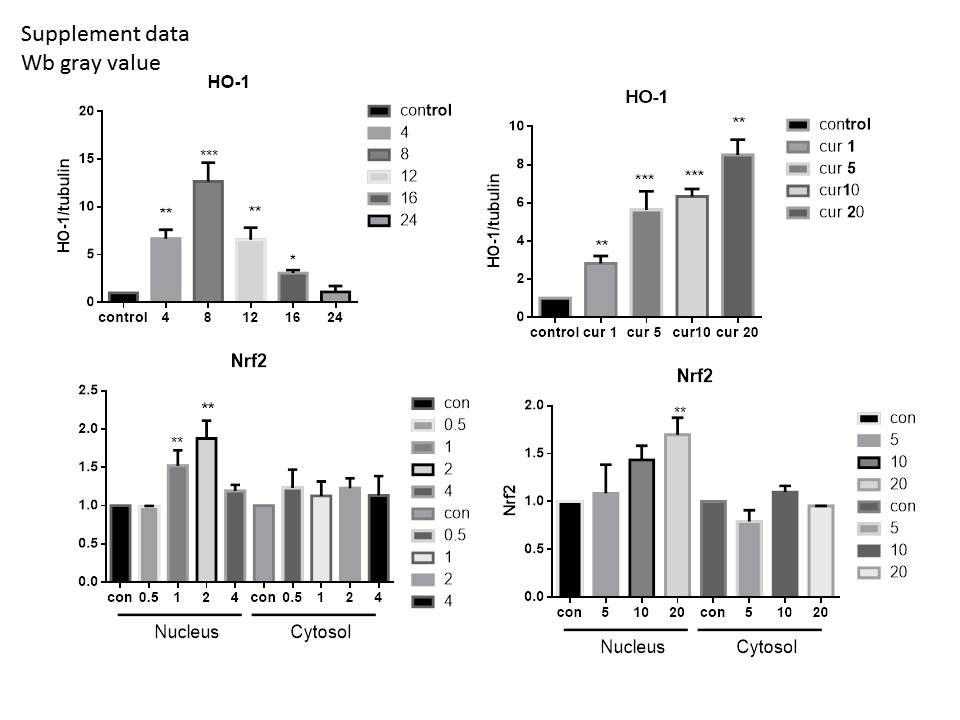

Supplement: Supplementary file 4 [file Image_3.JPEG]
